# Supplementary material for: Poly(methacrylate citric acid) as a Dual Functional Carrier for Tumor Therapy
Source: Pharmaceutics. 2022 Aug 24;14(9):1765. doi: 10.3390/pharmaceutics14091765 (PMC9506429; doi:10.3390/pharmaceutics14091765)
Supplement: Supplementary file 1 [file pharmaceutics-14-01765-s001.zip › pharmaceutics-1812172-supplementary.pdf]

Supplementary Materials

Poly(methacrylate citric acid) as a dual functional carrier for tumor therapy

Bo Yu[a], Yiping Shen[a], Xuejie Zhang[a], Lijuan Ding[a], Zheng Meng[a], Xiaotong Wang[a], Meihua Han[a], Yifei Guo[a,b,c,d],[*] Xiangtao Wang[a*]

[a.] *Institute of Medicinal Plant Development, Chinese Academy of Medical Sciences & Peking Union Medical College, No. 151, Malianwa North Road, Haidian District, Beijing 100193, China*

[b.] *Key Laboratory of Bioactive Substances and Resources Utilization of Chinese Herbal Medicine, Ministry of Education, Chinese Academy of Medical Sciences, Peking Union Medical College, Beijing 100094, China*

[c.] *Key Laboratory of New Drug Discovery Based on Classic Chinese Medicine Prescription, Beijing 100700, China*

[d.] *Beijing Key Laboratory of Innovative Drug Discovery of Traditional Chinese Medicine (Natural Medicine) and Translational Medicine, No. 151, Malianwa North Road, Haidian District, Beijing 100193, China*

Table S1. Zeta potential of the free drug without nanocarrier

Sample	RES	NIF	IBU	DOX	HCPT	CSL	HK	POD
ζ (mV)	10.5±1.1	11.1±0.7	15.5±0.9	39.2±0.9	-9.62±0.5	-13.5±1.3	-38.7±1.1	-8.43±0.4

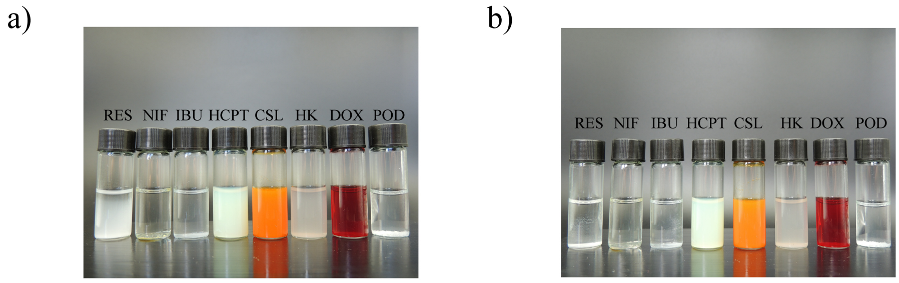

Figure S1. The images of drug-loaded nanoparticles: after preparation (a), storage for 24 h (b).

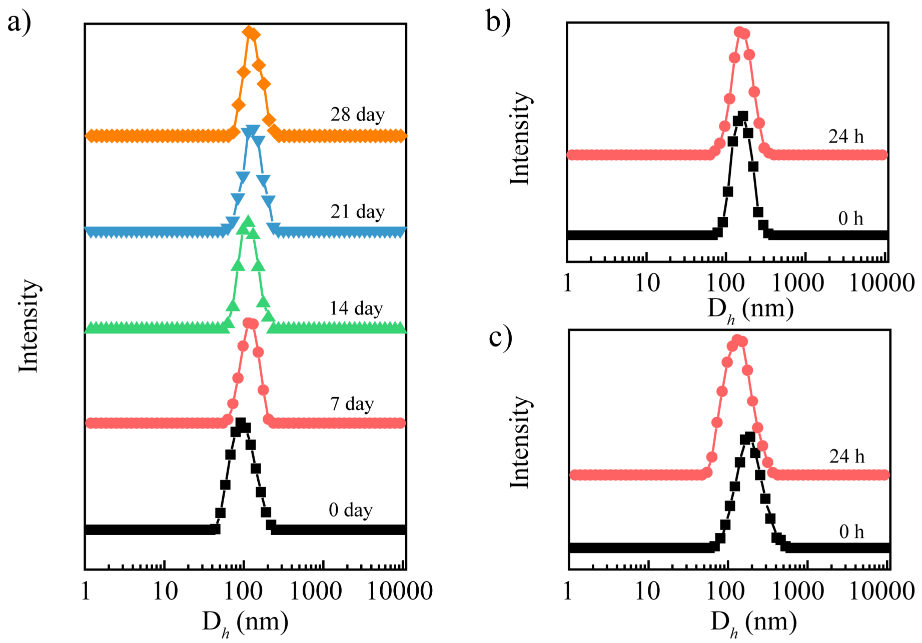

Figure S2. Particle size distribution curves of PCA/DOX NPs: storage stability at different times (a), glucose stability at different times (b), and plasma stability at different times (c).

Table S2. Drug release kinetic model for PCA/DOX NPs

Sample	pH	Zero-order model		First-order model		Higuchi model		Ritger-Peppas model	
		Equation	R^2	Equation	R^2	Equation	R^2	Equation	R^2
PCA/DOX NPs	5.5	$Q_t=0.46t+11.09$	0.902	$Q_t=63.46(1-e^{-0.03t})$	0.959	$Q_t=5.90t^{1/2}+0.54$	0.988	$Q_t=5.56(t^{0.52})$	0.985
PCA/DOX NPs	7.4	$Q_t=0.40t+5.73$	0.974	$Q_t=70.72(1-e^{-0.01t})$	0.963	$Q_t=4.86t^{1/2}-2.37$	0.983	$Q_t=3.36(t^{0.57})$	0.984

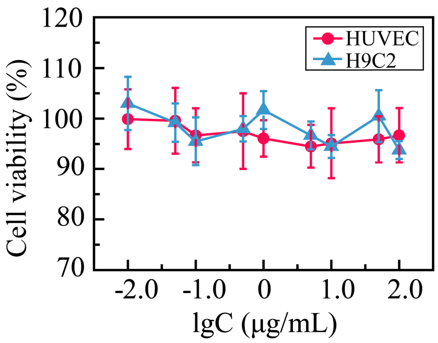

Figure S3. Cell viability of PCA against normal HUVEC and H9C2 cell lines for incubation 48 h at 37 °C, n = 5.

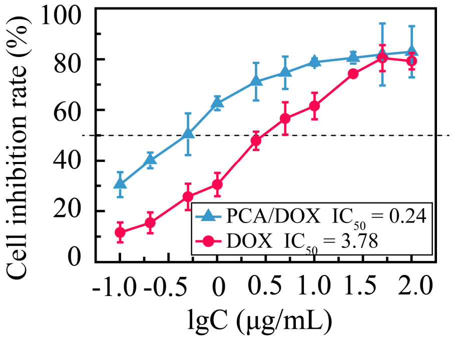

Figure S4. Cell viability of PCA/DOX NPs against 4T1 cell line for incubation 72 h at 37 °C, n = 5.
